# The effect of different kinematics on apical debris extrusion with a single-file system

**DOI:** 10.1007/s10266-023-00802-3

**Published:** 2023-03-14

**Authors:** Taher M. N. Al Omari, Giusy Rita Maria La Rosa, Rami Haitham Issa Albanna, Abedelmalek Tabnjh, Flavia Papale, Eugenio Pedullà

**Affiliations:** 1 Department of Conservative Dentistry, Faculty of Dentistry, Jordan University of Science and Technology, Irbid, Jordan; 2 Department of General Surgery and Medical-Surgical Specialties, University of Catania, Catania, Italy; 3 Department of Applied Dental Science, Faculty of Applied Medical Science, Jordan University of Science and Technology, Irbid, Jordan

**Keywords:** Adaptive motion, Canal pro Jeni, Debris extrusion, One Curve, Optimum Torque Reverse

## Abstract

To compare the amount of extruded debris caused by different motions using a single-file system. Fifty mandibular first molar teeth were randomized into 5 groups (*n* = 10) according to the motion tested: Optimize Torque Reverse (OTR), TF Adaptive Motion (TFA), continuous rotation (CR), reciprocation motion (+ 150°, −30°) (REC), and Jeni motion (Jeni). One Curve single file 25/06 (Micro-Mega, Besançon, France) was used in all experimental groups. The root canals were irrigated with 2.5% NaOCl, and the extruded debris were collected at pre-weighted glass vials. The glass vials were kept inside an incubator for one week at 70 °C to dry out the irrigating solution. The extruded debris was quantified by subtracting the pre-instrumentation from the post-instrumentation weight of the glass vials. The time required for each instrumentation procedure was digitally recorded. All data were analyzed statistically with one way ANOVA and post hoc Tukey test (*P* < 0.05). All the motions extruded apically debris with Jeni mode caused significantly less debris extrusion than TFA, REC, and CR (*P* < 0.05) while no significant difference emerged with OTR. Preparation time was not significantly different in all groups. Within the limits of the present study, all the kinematics produced apically debris extrusion, with Jeni reporting a similar amount of debris compared with OTR and significantly less than TFA, REC, and CR. Preparation time was similar among the tested kinematics.

## Introduction

Debridement, disinfection, and obturation of the root canals are crucial elements for the success of endodontic therapy. These procedures may lead to the apically debris extrusion causing a possible periapical inflammation, post-operative pain, flare-up, or delay of periapical healing [1, 2].

Although nickel–titanium (NiTi) rotary instruments reduced the debris extrusion compared with manual files [3, 4], all file systems generated it. Thus, manufacturers proposed several innovations in terms of kinematics and NiTi instrument to guarantee a satisfactory and as well as possible fast canal preparation [1]. One Curve (OC) (Micro-Mega, Besançon, France) is a single-file canal-shaping, with ISO tip size of 25 and a constant taper of 6% at various cross sections, with a triangular shape at the tip and an S-shape close to the shaft. These geometrical features should improve the coronal debris expulsion [2].

The effect of kinematics on apically extrusion debris is controversial. Arslan et al. reported that reciprocating motion (REC) extruded considerably less debris than continuous rotation (CR) [5], while Bürklein et al. asserted the contrary [6]. The introduction of new hybrid kinematics like TF Adaptive (TFA), Optimum Torque Reverse (OTR), and Canal Pro Jeni (Jeni) has been suggested to decrease the incidence of file separation and enhance the file progress inside the canals [7–9]. TF Adaptive (KaVo Kerr, Brea, CA) is a complex motion that changes the rotation angle according to the loading on the file and rotates free with a fracturing of second stop after 600-degree clockwise rotational angle [10]. Optimum Torque Reverse is a motion introduced in 2015 [11]. It allows the file to rotate freely in clockwise rotation until a load point; then, the file rotates in reciprocating motion with angles 180° CW and a 90° CCW [8, 11].

Canal Pro Jeni is a new motor introduced in early 2020 by Coltene (Coltène/Whaledent, Altstätten, Switzerland). It incorporates the patented digital assistance system for canal preparation (Jeni motion) with the aim to minimize the risk of file breakage [9]. This smart motion assesses the torsional stress level on the file reading the current intensity needed to move the file and adjust accordingly the instrument motion. The adjustment regards continuous and different changes of several movement parameters (i.e., direction, angles, and speed) allowing safe and predictable preparation.

Reciprocation and rotational motion were studied extensively with regards of debris extrusion [1, 12–15]; however, few or no studies reported the effect of new motions like TFA, OTR, and Canal Pro Jeni on the incidence of apically debris extrusion.

Therefore, this study was designed to compare the amount of apically extruded debris caused by Optimum Torque Reverse, Canal Pro Jeni, Reciprocation, TF Adaptive motion, and Continuous Rotation using One Curve single-file system. The null hypothesis was that there would be no difference in terms of preparation time and the amount of apically extruded debris between the different motions.

## Materials and methods

### Sample size calculation

With a sample size of 50, a number of groups of 5, a power of 0.90, and *α* = 0.05, the present sample size was adequate to detect a minimum effect of *f* = 0.585, which is considered a large effect. The analysis was performed with G*Power 3.1 for Macintosh (Heinrich Heine, Universität Düsseldorf, Dusseldorf, Germany).

### Teeth selection

Fifty mandibular first molars extracted for periodontal or extensive caries reasons were allocated for the study. Teeth were preserved in physiological saline solution until use. Samples were prepared and cleaned from soft tissue remnants on the surface with ultrasonic tip. Mesial roots were separated with a diamond disk (Komet Italia Srl, Milan, Italy). The presence of two separate canals and two separate apical foramina was confirmed with digital periapical radiographs in both mesiodistal and buccolingual directions [16, 17]. The roots with similar measurements at buccolingual and mesiodistal directions, closed apices, uncalcified canal and moderate to severe curvatures (25°–40°) were selected for the study [18]. Exclusion criteria included calcifications, straight canals, root resorption, apical minor constriction greater than a size #25 K-file, prior root canal treatment, crack, and excessive tissue loss.

Access to the canals was performed with diamond burs (Intensiv, SA, Montagnola, Swiss) and root lengths standardized to 18 mm by terming the cusp until flat reproducible point. The working length (WL) was determined under surgical microscope using a #10 K file (Dentsply Maillefer, Ballaigues, Swiss) to the root canal terminus and deducting 1 mm. Canal patency was controlled with a size 15-K file [19]. Then, the teeth were numbered and divided randomly into 5 groups (*n* = 10 each) according to the motion tested (Available at: www.random.org).

### Experimental design

The debris collection apparatus was based on a previous study by Myers and Montgomery [20] with some modifications. Fifty empty glass vials were weighed five times by an electronic balance (Citizen CX 220 Analytical Lab Balance, Internal Cal. Weighing Hook, USA) with an accuracy of 10^–4^ and their mean values were recorded. Each root was fixed to the glass vial opening by a custom-made rubber stopper of addition silicon material that ensured a seal. The root tip was submerged in the glass vial and A 23 G irrigation needle placed into the rubber stopper to equalize the inner and outer pressure. The glass vial firmly attached to the base of a larger outer glass container, which was filled with a controlled temperature of 37 °C water bath, as confirmed with an electrode thermometer (MN35, Digital Mini MultiMeter, Boston, Massachusetts, USA). The surface of the glass vial was covered with aluminum foil to shield the view of the root apex during instrumentation.

### Shaping procedures

Initial root canal preparation was performed using size 15 K-files to create a glide path for each root canal [21]. All teeth were prepared by one operator under magnification (Zumax Medical Co, Ltd, Suzhou, Jiangsu, China), while the assessment of debris was performed by a second examiner who was blind with respect to all experimental groups. The operator was an experienced endodontist who had intensive experience with the use of each of the four kinematics. All teeth were prepared using One Curve single-file instrument with slow pecking motion until reach the full working length. Samples were divided in 5 groups according to the kinematics used for shaping procedure (*n* = 10) for each group.

OTR group: Tri Auto ZX2 motor (Morita Corp, Japan) was used on OTR motion. For the OTR motion speed was adjusted to 300 rpm, the 180° rotation angle was selected, and the trigger torque was set to 0.2 N·cm.

Jeni group: Instruments were activated by the preparation program for One Curve instruments in Canal Pro Jeni motor. This kinematic applied to the instrument the automatic and unchangeable Jeni motion performing a starting clockwise full rotational motion automatically changed for direction angles and speed according to the torque and current intensity needed to move the file in apical direction.

TFA group: Adaptive motion was applied using TF Adaptive preset and unchangeable program on Elements Motor (SybronEndo, Glendora, CA, USA).

REC group: Partial reciprocation with rotational effect in clockwise direction using 150°in CW and 30° in CCW at 300 rpm and 3.5 Ncm torque was used on E-xtreme motor (Eighteeth, China).

CR group: Clockwise Rotational motion at 300 rpm and 3.5 Ncm torque was used on Elements Motor (SybronEndo).

Each sample was gentle irrigated with a total of 10 mL of 2.5% sodium hypochlorite (NaOCl) at environmental temperature using a side-vented 27-G needle (Endo-Eze Irrigator, Ultradent Products, South Jordan, UT, USA) which was marked with a rubber stopper placed at 2 mm short of the WL, and a high vacuum suction was used to evacuate excess irrigation. Irrigation was performed at four stages of the procedure: 2.5 ml of NaOCl was used for irrigation before insertion of the instrument into the canal, 2.5 ml after the first withdrawal of the file (for cleaning), 2.5 ml after reaching WL and 2.5 ml after completing the instrumentation [22]. At the end of the procedure, the outer root surface was irrigated with 1 mL bi-distilled water inside the glass vial to collect any residual debris on the external root surface. The preparation time was recorded in seconds by a digital chronometer (Digimatic; Mitutoyo Co, Kawasaki, Japan). The starting point was the first insertion of the file into the canal, and the end point was the end of the final irrigation [22].

The glass vials were transferred into incubator for one week at 70 °C to dry out the irrigating solution. Later after the dry out of the irrigation solution, the glass vials were weighted five times using the same analytical balance and the mean value was calculated. Then, the weight of extruded debris in mg was calculated by the difference between pre- and post-weights.

### Statistical analysis

The SPSS software (V. 28, State College, PA, USA) was used. One-way ANOVA and post hoc Tukey tests were applied with *P*-value set < 0.05.

## Results

The mean weight (standard deviation) of the amount of apically extruded debris and the preparation time (s) for each group are shown in Table 1. Jeni showed significantly lower apically extruded debris compared with TFA, REC, and CR (*P* < 0.05) while no significant difference emerged with OTR (*P* > 0.05). No statistically significant difference emerged among OTR and the other tested motions neither among TFA, REC, and CR (*P* > 0.05). The preparation time was not significantly different among the all tested groups (*P* > 0.05).

**Table 1:** The mean and standard deviation (SD) of the weight of apically extruded debris and preparation time in the groups

Group	Debris extrusion (mg) Mean (SD)	Preparation time (s) Mean (SD)
Jeni	7.803^a^(1.037)	178^a^ (35)
OTR	8.297^ab^(1.383)	175^a^(41)
REC	9.450^b^ (2.588)	171^a^(31)
TFA	9.611^b^(0.567)	165^a^(38)
CR	9.890^b^(1.186)	124^a^(35)

*OTR* Optimum torque reverse, *REC* reciprocating motion, *TFA* TF adaptive, *CR* continuous rotation

Different superscript letters ^a^,^b^ indicate statistically significant differences among the groups (*P* < .05)

## Discussion

The aim of this in vitro study was to evaluate the amount of apically extruded debris using One Curve single file with five different kinematics. The same instrument was used for all kinematics to standardize the groups and exclude any other variables which could have impacted the findings. One Curve is a single rotary file for single use [23]. One Curve is a file designed for continuous rotation with three cutting angles at the tip and two close to the shaft. This geometry should improve the coronal debris transportation and reduce their apical accumulation [2, 24, 25]. To date, only few studies compared the extrusion of debris of One Curve with that of other NiTi instruments [2, 26–28]. In addition, to the best of our knowledge, no previous studies compared the all five-kinematics tested in terms of apically extruded debris.

The amount of extruded debris was collected using the Myers and Montgomery approach which ensures to collect small amount of material [29]. On the other hand, this method presents some disadvantages, one of the most important of which is the lack of simulation of pulp or periapical tissues which may act as barriers restricting the quantity of extrusion in vivo [29]. For this reason, some authors have proposed to use agar gel or foam to simulate periapical tissues [30]. Yet, the foam could absorb the irrigant solutions and debris influencing the findings [28] and for this reason, it was not applied.

Sodium hypochlorite was used as irrigant to simulate clinical conditions; however, this procedure can also entails the formation of sodium hypochlorite crystals which increases the weight of the collected apically extruded debris [29]. This is the reason why the weight of apically extruded debris was major compared to other studies using distilled water [31].

All tested kinematics produced apically extruded debris in some degree. Jeni motion caused significant less debris extrusion compared to TFA, REC, and CR while a similar amount compared with OTR. No other significant differences emerged among the OTR and the other groups neither among TFA, REC, and CR. Preparation time was similar in all experimental groups. Thus, the null hypothesis can be partially rejected.

OTR and Jeni are new kinematics recently released, therefore no data about apical debris extrusion during shaping procedure have been reported in literature; only one retreatment study evaluated the extrusion of debris with Jeni motion compared with continuous rotation [9].

The OTR motor rotates constantly in a clockwise manner under a predetermined torque value in the OTR mode. When the torque surpasses this amount, the rotation switches to an alternating 90° counterclockwise and 180° clockwise spin until the torque is reduced to the specified level [32]. Jeni motion constantly measures parameters, such as pressure, torque, tension, or electrical intensity, by means of an automatized algorithm and adapts the type of file movement accordingly [9]. The similar amount of debris generated by Jeni and OTR could be due to the hybrid nature of kinematics. In addition, Jeni performed better than TFA, REC, and CR probably due to the continuous alternation of angles and speed which results in improved efficiency and consequently less apical extrusion.

No significant difference emerged among the other kinematics. Continuous rotation, which acts like a screw conveyor, seems to promote coronal transportation of dentine chips and debris whereas reciprocal motion appeared to boost debris transportation toward the apex [33]; conversely, De-Deus et al. reported a greater quantity of apically extruded debris with conventional multi-file rotary system compared to reciprocating [34]. Other studies did not show significant differences between the two kinematics, in agreement with our results [31, 35]. The different findings are probably to the different methodological conditions including file system and angles of reciprocation applied.

In agreement with our results, debris extrusion generated by TF Adaptive was not significantly different from 150° CW/30° CCW reciprocation or continuous rotation [36]. Conversely, another study reported that WaveOne Gold reciprocating single-file system was associated with less extrusion of debris compared with the Twisted File Adaptive system [37]. As mentioned above, the studies are not direct comparable because of the different methodology.

The time results are probably explained by the standardized procedure applied to all groups which guaranteed to uniform preparation times. It is important to emphasize that this is the first study evaluated the impact of automatic kinematics, such as Jeni and OTR, on the apical debris extrusion generated during a primary root endodontic treatment. In addition, the use of sodium hypochlorite rather than distilled water allowed to provide more clinically relevant information. The findings are clinically interesting because they show that hybrid motions did not slow down the procedure in significantly manner compared to the other kinematics.

Clinicians should be cautious because laboratory conditions may represent a limitation and tooth anatomy could impact differently the apically extruded debris generated in vivo. Furthermore, the present study was conducted using only mesial roots of mandibular first molars with no apical resorption. Apical resorption is frequent in primary teeth [38] and its effect on apical extrusion debris should be further examined. In addition, the time necessary to complete the root canal treatment of two/three rooted primary molars may be longer than that of a single mandibular mesial canal. In addition, this study evaluated debris only in quantitative terms and no qualitative analysis was performed.

Further studies are required to better understand the effects of new kinematics on apically extruded debris focusing also on the clinical outcomes such as post-operative pain.

## Conclusions

Under the current limitations, all the tested kinematics produced apical debris extrusion. Jeni mode showed a similar apically debris extrusion with OTR and significantly less than TFA, REC, and CR. No significant differences emerged among OTR and the other kinematics neither among TFA, REC, and CR. Preparation time was similar in all groups.

## Data Availability

The datasets generated during the current study are available from the corresponding author on reasonable request.
